# Myocardial perfusion SPECT in Germany from 2012 to 2021: insights into development and quality indicators

**DOI:** 10.1007/s00259-023-06129-z

**Published:** 2023-02-13

**Authors:** O. Lindner, W. Schäfer, C. Rischpler, S. Silber, W. Burchert, on behalf of the Working Group “Cardiovascular Nuclear Medicine” of the German Society for Nuclear Medicine

**Affiliations:** 1 Institut Für Radiologie, Nuklearmedizin Und Molekulare Bildgebung, Herz- Und Diabeteszentrum NRW, Bad Oeynhausen, Germany; 2 Klinik Für Nuklearmedizin, Kliniken Maria Hilf GmbH, Mönchengladbach, Germany; 3 Klinik Für Nuklearmedizin, Universitätsklinik Essen, Stuttgart, Germany; 4 Abteilung für Nuklearmedizin, Klinikum Stuttgart, Stuttgart, Germany; 5 Kardiologie Zentrum München, Munich, Germany

**Keywords:** Myocardial perfusion imaging, Survey, Utilisation review, Utilisation statistics

## Abstract

**Purpose:**

This paper summarises the results of 4 national surveys on the numbers, utilisation and technique of myocardial perfusion SPECT (MPS) from 2012 to 2021.

**Methods:**

A one-page questionnaire for information on MPS in 2012, 2015, 2018 and 2021 was sent to German centres practising nuclear medicine. To check for representativeness, the numbers obtained were related to official annual data and furthermore to the numbers of invasive coronary angiography procedures (ICA).

**Results:**

MPS examinations increased by > 40% from 2012 to 2021 and showed a centralisation with increasing MPS per centre. In 2020, a mild impact of the COVID-19 pandemic could be observed in the form of only a slight MPS increase, which was compensated in the following year. Outpatient care cardiologists represent the most important referrer (70%). Mostly, 2-day protocols were used. One-day protocols and stress-only protocols showed insignificant changes. The use of exercise stress decreased steadily. In 2021, exercise stress was replaced by pharmacological stress as the most frequent stress modality. Camera systems showed a shift to more SPECT-CT systems. The use of gated SPECT increased to almost 90%. Quantitative scoring showed an increasing acceptance. The ratio of invasive coronary angiographies (ICA) to MPS was between 3.9 and 4.5. A significant proportion of ICA in the context of CCS (chronic coronary syndrome) was performed without prior testing for ischaemia.

**Conclusion:**

The 2012 to 2021 MPS surveys reveal a continuously growing number of examinations with only a mild temporary effect of the COVID-19 pandemic and a centralisation with increasing numbers per centre. Performance and technical data reveal a high-grade adherence of MPS practice to the current ESC guideline. A large potential of non-invasive diagnostics remains for the future.

## Introduction

The working group Cardiovascular Nuclear Medicine of the German Society of Nuclear Medicine performs regular surveys for information on development, utilisation and technique of myocardial perfusion SPECT (MPS) in Germany. The first survey started in 2006 and has been carried out every 3 years since 2009 [1–5].

Compiled data from the years 2005 to 2012 were published in 2014 in this journal [6]. Together with the recent survey from the year 2021, this paper summarises the development from 2012 to 2021. Additionally, the 2021 survey provides some insight into the impact of the COVID-19 pandemic on MPS examinations.

Even though these are only national progression and performance figures, the data may allow limited conclusions to be drawn about the general development of MPS imaging in Europe. Long-term European survey data are rare. The last published survey data refer to the years 2005 and 2007 [7].

## Methods

Based on the register of members of the Germany Society of Nuclear Medicine, physicians and sites practising nuclear medicine were identified and updated before every survey. A one-page survey form with a cover letter was faxed to the head of each centre in January 2013, 2016, 2019 and 2022 to obtain information from the previous year in each case. In the absence of a response, a first reminder was forwarded after 4 weeks and a second 4 to 6 weeks later, in some cases after personal contact. The surveys were closed at the end of May.

The one-page questionnaire comprised the following items in all surveys:

- Number of MPS patients
- Number of stress and rest MPS examinations
- Number of different types of stress test
- Number of patients by study protocol
- Percentage of patients examined with gated SPECT
- Percentage of patients examined with attenuation correction (AC)
- Type of AC
- Usage of semiquantitative scoring (categories: never, always, intermediate (= between “never” and “always”))
- Type of camera
- Percentage referrals from cardiologists, primary care physicians, from in-patient ward physicians, and others
- Changes in referral (categories: no change, unchanged, more, unknown) and, in case of decline, potential reasons (categories: stress echocardiography, cardiac CT, cardiac MRI, invasive coronary angiography (ICA), and in 2021 COVID-19 pandemic)

In order to verify the representativeness of the survey and to estimate the total number of MPS examinations with sufficient reliability, the figures obtained were related to the data of the National Association of Statutory Health Insurance Physicians (NASHIP) (Kassenärztliche Bundesvereinigung (KBV), www.kbv.de). They represent the official MPS examination numbers of non-hospitalised patients with statutory health insurance. The NASHIP data were annually communicated after written request by the German Society of Nuclear Medicine.

Estimation of the total number of MPS has been described in detail elsewhere [6].

Additionally, the MPS numbers were related to the ICA and intervention data of the respective official German cardiology report (Deutscher Herzbericht) [8–11]. At the time of writing this manuscript, the most recent data of the cardiology report referred to 2020. Therefore, the 2021 MPS data could only be related to these ICA and intervention data.

### Statistical analysis

Statistical analysis was performed using SPSS Statistics 27 (IBM, Chicago, IL, USA).

For analysis of changes in the four queries, the Kruskal–Wallis test by rank with pairwise comparisons and Bonferroni correction was used.

Pearson’s chi-squared test was applied to the categorical data of the items changes in referral and semiquantitative scoring.

A *P* value of < 0.05 was considered statistically significant.

## Results

### MPS patient numbers and data from cardiology

Table 1 lists the numbers of MPS patients recorded in the surveys, the estimated total number of patients after adjustment with the NASHIP statistics, and data of the participating centres. Based the NASHIP data, clearly more than 50% of all MPS were included in all surveys.

**Table 1:** MPS procedures

	2012	2015	2018	2021
MPS patients (survey)	105,941	121,939	145,930	133,057
% total MPS patients	61	64	67	54
Total MPS patients*	173,941	190,530	217,806	246,402
Number of centres	278	268	291	218
Mean MPS/centre	381	455	502	610
< 50 MPS/year	21%	18%	15%	11%
> 1000 MPS/year	10%	12%	15%	18%

*Estimate based on the NASHIP (National Association of Statutory Health Insurance Physicians) statistics

**Fig. 1:**
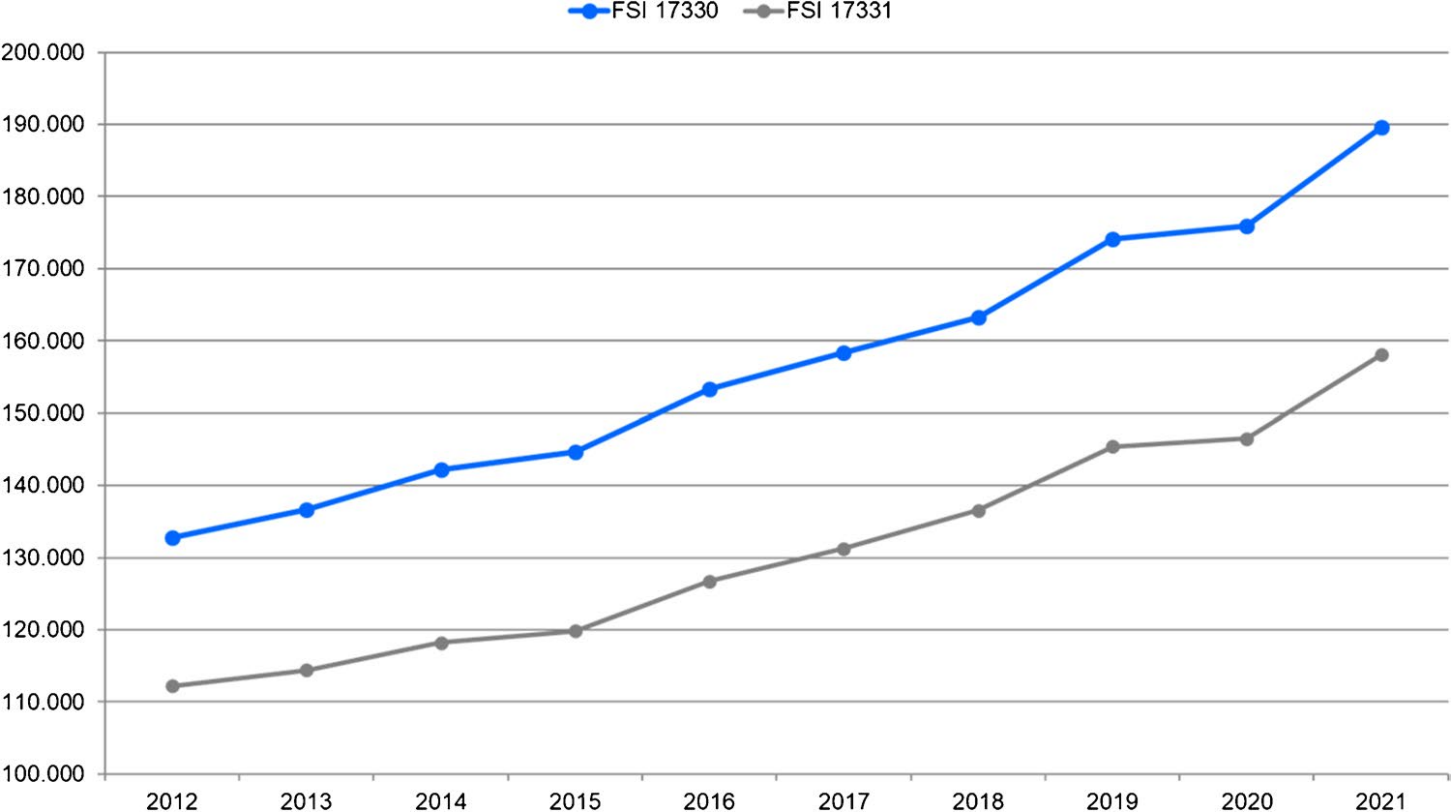
MPS examinations of non-hospitalised patients with statutory health insurance according to the National Association of Statutory Health Insurance Physicians (Kassenärztliche Bundesvereinigung) for the fee schedule items (FSI) 17,300 (stress MPS) and 17,331 (rest MPS) from 2012 to 2021

Figure 1 shows the time course of the NASHIP counts of the fee schedule items 17,330 (stress MPS) and 17,331 (rest MPS) from 2012 to 2021. The item stress MPS increased by 43% and the item rest MPS by 41%.

During the period under review, mean and median MPS examinations per centre increased substantially. There was a decrease in centres with < 50 MPS/year (< 1 MPS/week), and, on the other hand, a continuous increase in those with > 1000 MPS/ (> 4 MPS/d) over time (Table 1).

A total of 110 centres provided data to all surveys between 2012 and 2021. They demonstrated a 47% increase in their MPS patients from 59,728 (2012) to 87,973 (2021).

The number of ICA, interventions, and ratios is shown in Table 2. ICA figures and the ICA/MPS ratio were decreasing after 2015. Revascularisation numbers varied with those of ICA. Their ratio remained constant.

**Table 2:** Invasive coronary angiographies (ICA), interventions and MPS ratios

	2012	2015	2018	2021
ICA	857,688	911,841	867,138	798,751*
ICA/MPS	4.0	4.5	4.0	3.2
Revascularisation (total)	392,473	416,979	411,110	371,357*
PCI	337,171	365,038	366,840	333,373*
Bypass	55,302	51,941	44,270	37,984*
Revascularisation/ICA	0.46	0.46	0.47	0.46

*Data refer to 2020. More recent data not available at the time of writing the manuscript

### Changes in referral behaviour and competitive methods

In all surveys, participants were asked to assess their individual development of MPS examinations and, in case of a decline, the causally suspected competitive modality or modalities.

**Fig. 2:**
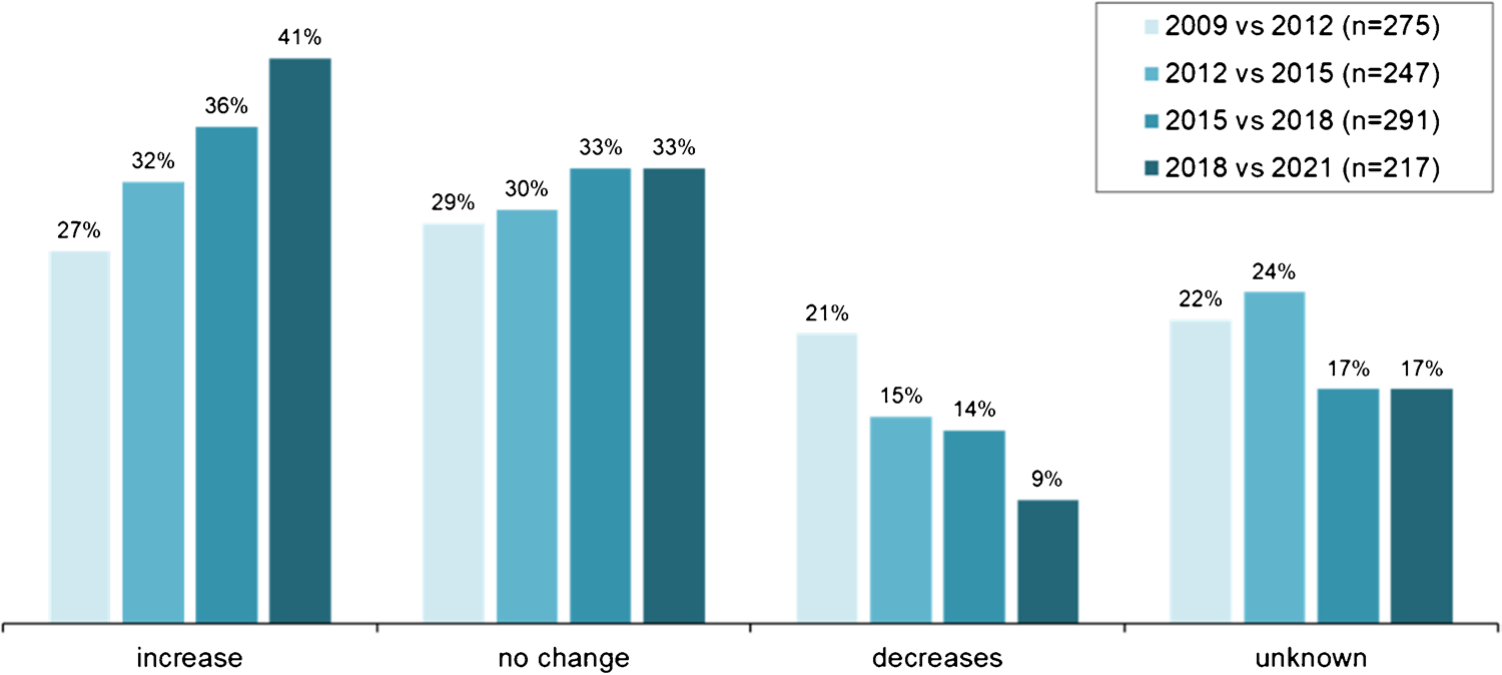
Changes in MPS referral (individual estimates of the participating centres)

The assessments from 2012 to 2021 varied significantly (*P* < 0.001) and are depicted in Fig. 2. Since 2012, there has been a steady increase in centre with rising MPS examinations and a constant proportion of institutions with unchanged numbers. Correspondingly, the proportion of centres with declining numbers decreased.

**Fig. 3:**
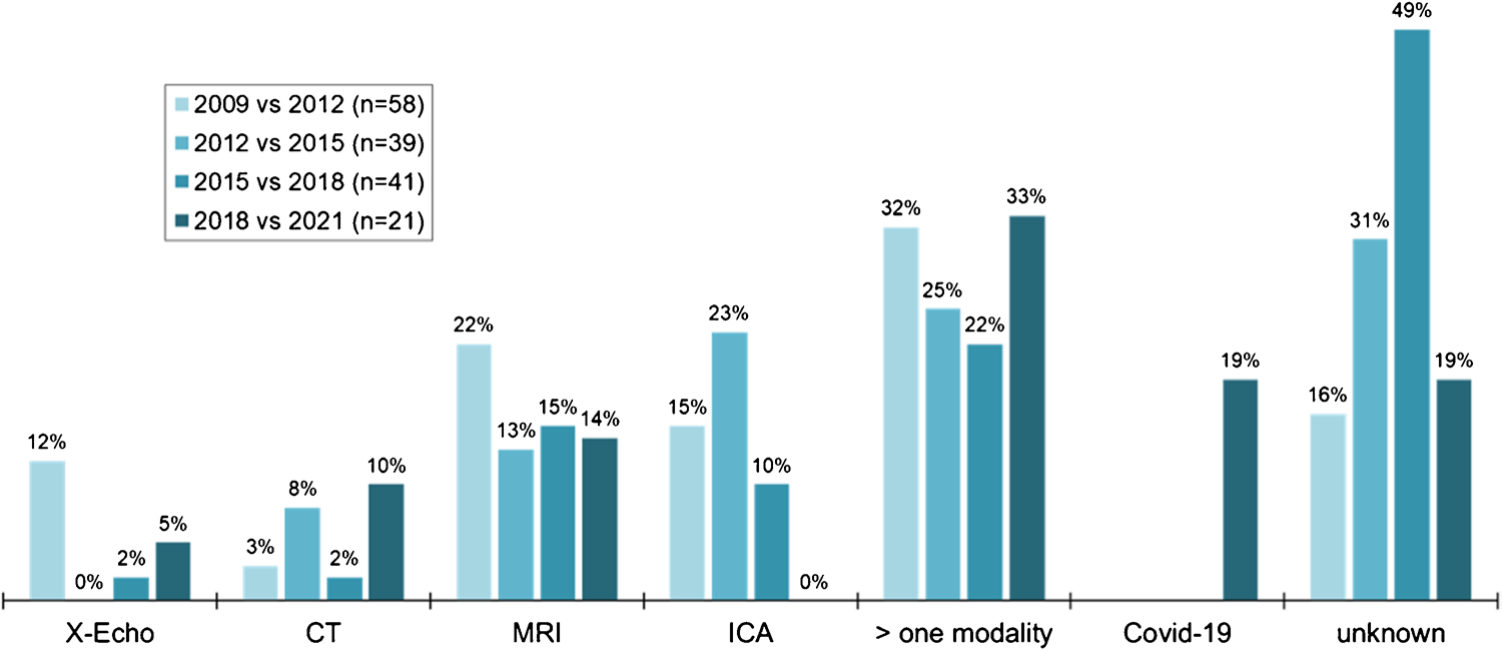
Possible reasons for declining MPS examinations

The presumed reasons for fewer MPS patients are shown in Fig. 3. A single dominant competitive modality was not discernible. All in all, the other imaging methods represented the greatest competition. The COVID-19 pandemic only played a minor role.

### MPS referrals

**Fig. 4:**
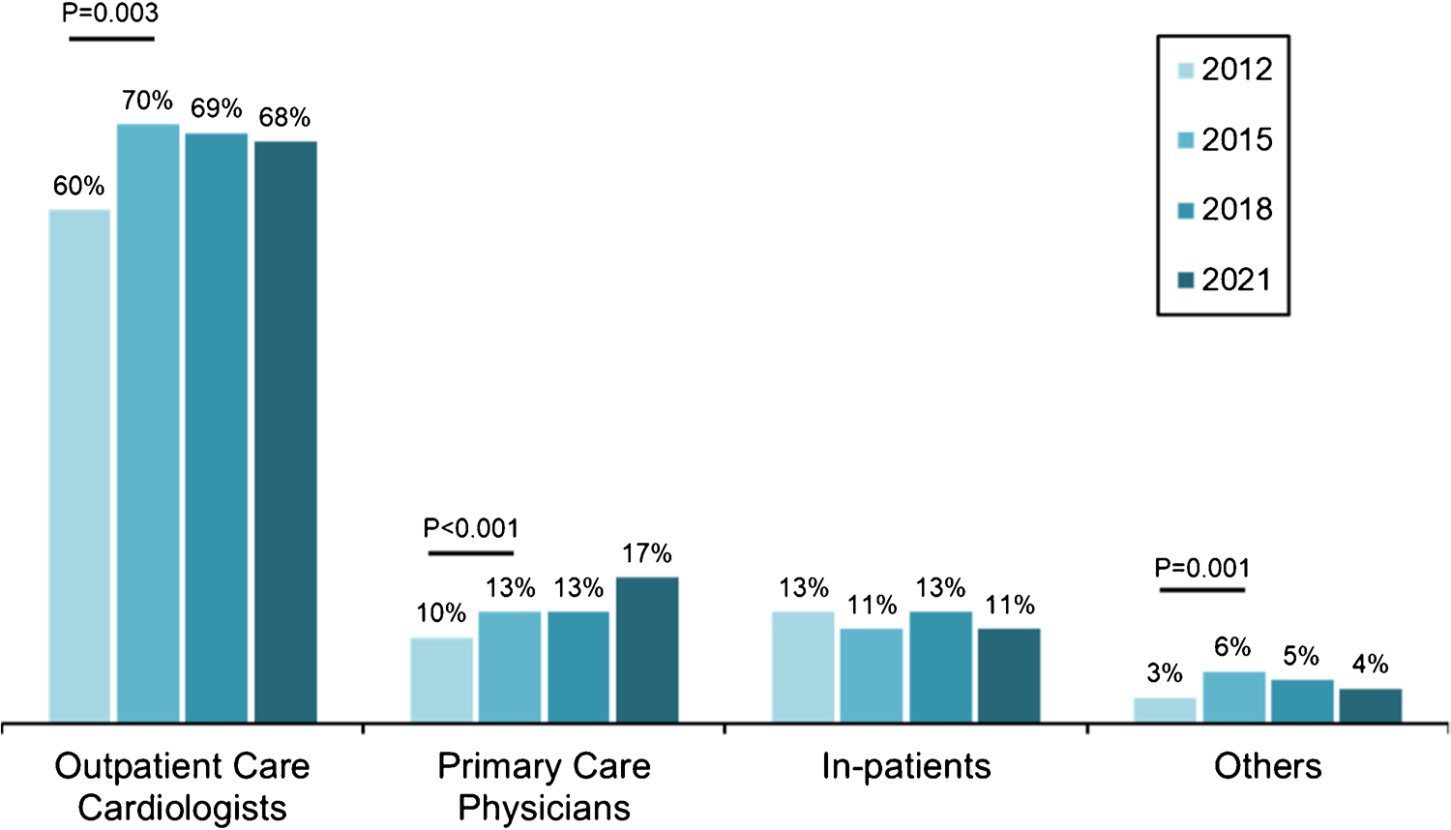
MPS referral structure from 2012 to 2021

The MPS referral structure is illustrated in Fig. 4. Outpatient care cardiologists represented the most important referral group. They showed a significant increase from 2012 to 2015 (*P* = 0.003) and a constant proportion in the further course. Primary care physicians showed a mild increase which was significant from 2012 to 2015 (*P* < 0.001). Referrals from other physicians and from in-patient ward physicians mildly fluctuated. Formally, there was a significant increase in the group of other physicians from 2012 to 2015 (*P* = 0.001).

### MPS study protocols

**Fig. 5:**
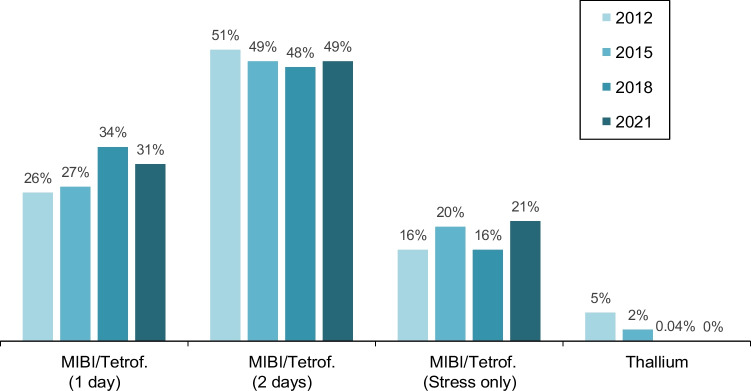
MPS protocols from 2012 to 2021. Changes between the surveys were not significant

Utilisation of the different MPS protocols is depicted in Fig. 5. In the surveys, Tc-99 m-MIBi or Tc-99 m-tetrofosmin were not asked separately.

Mostly, 2-day protocols were used. Over time, only insignificant changes with a mild increase in 1-day (stress and rest) and stress-only protocols could be observed. Thallium has been abandoned since 2018.

### Stress techniques

**Fig. 6:**
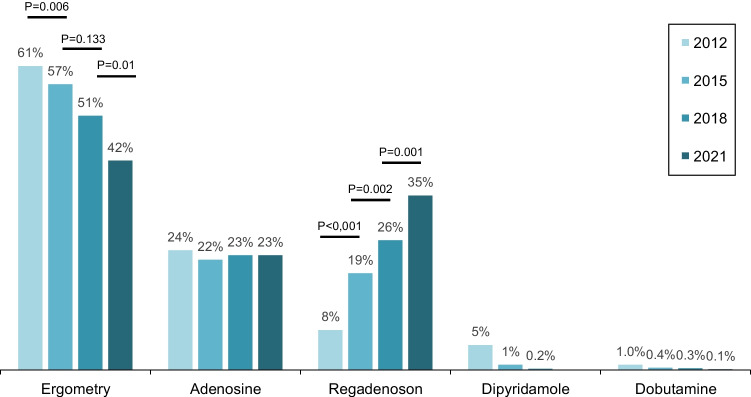
MPS stress testing from 2012 to 2022

Figure 6 shows the utilisation of the different stress techniques. The use of exercise stress decreased steadily. Formally, the decline was only insignificant from 2015 to 2018 (2012–2015, *P* = 0.006; 2015–2018, *P* = 0.133; 2018–2021, *P* = 0.01). In 2021, exercise stress was replaced for the first time by pharmacological stress as the most frequent stress modality. Regadenoson showed a rapid and significant increase (2012–2015, *P* < 0.001; 2015–2018, *P* = 0.002; 2018–2021, *P* = 0.001) and has been ahead of adenosine since 2018. The adenosine proportion remained constant. Dipyridamole is not licensed in Germany as an MPS stressor and was no longer queried in 2021. Dobutamine as a 2nd choice stress agent was used in very rare cases with a declining proportion.

### Camera systems and attenuation correction

**Fig. 7:**
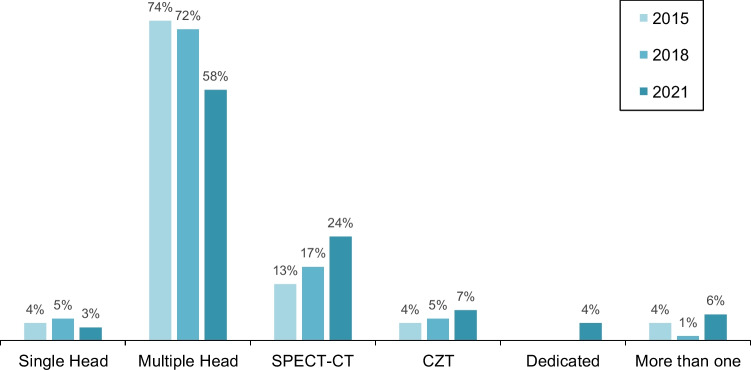
Camera systems for MPS from 2015 to 2021 by centres

Camera systems (Fig. 7) and attenuation correction data (Table 3) were available from 2015 to 2021. Statistics of the camera systems used revealed significant differences (*P* = 0.019) over time. Single-head cameras were still utilised in a few centres (3%). In 2021, they examined only 1.2% of the patients. The number of centres with SPECT-CT systems was steadily growing, whereas those with CZT systems increased only slightly. The latter performed about 19% of all MPS in the queries. Centres with dedicated cardiac cameras or with more than one camera system for MPS imaging were rather the exception.

**Table 3:** Attenuation correction in MPS

	**2015**	**2018**	**2021**
Prone/supine imaging	11%	10%	8%
Transmission sources	6%	3%	1%
CT-based AC	11%	20%	30%
> one AC method available	1%	1%	1%
No attenuation correction	71%	66%	60%
Patients studied with AC*	25%	26%	33%

The values in the upper 5 lines refer to the number of responding centres

*Changes between the surveys were not significant

The number of centres using attenuation correction for MPS imaging was increasing. In 2021 at least one-third of all MPS patients were studied with an attenuation correction procedure. Statistically the chances were not significant (*P* = 0.175). The main attenuation correction method was CT. Transmission sources for attenuation correction were decreasing and played a marginal role in the last survey.

### Gated SPECT and quantitative scoring

The proportion of MPS patients acquired with gated SPECT instead of ungated SPECT was increasing steadily, most recently reaching nearly 90% (Table 4). Statistically the changes were not significant.

**Table 4:** MPS imaging as *gated* SPECT

	**2012**	**2015**	**2018**	**2021**
Gated stress	73%	80%	86%	89%
Gated rest	70%	78%	87%	88%
Gated both	67%	76%	83%	87%

Data represent percentages of MPS patients with *gated* SPECT. Changes between the surveys were not significant

The percentages of centres performing a regular, an intermediate or no quantification of myocardial perfusion with scores are listed in Table 5.

**Table 5:** Utilisation of perfusion scores

	**2012**	**2015**	**2018**	**2021**
Regular	35%	53%	67%	72%
Intermediate	23%	23%	17%	15%
Never	41%	24%	16%	13%

Data represent percentages of centres

The data show an increasing acceptance (*P* < 0.001), with a doubling from 2012 to 2021. Nevertheless, a low proportion (13%) of centres not scoring remained. In 2021, they examined only 6.4% of the patients.

## Discussion

The present paper compiles the results of four German MPS surveys from 2012 to 2021 and provides an overview of the development of MPS imaging by patient numbers and technique. The survey data were cross-checked with the official NASHIP figures and revealed that clearly more than 50% of all MPS were covered in all surveys. Thus, the results found can be considered representative. Furthermore, the high response rate of such a survey confirms that our long-term concept of a 3-year survey with an easy-to-use one-page questionnaire is widely accepted and may serve as a template for similar projects.

Our data only include myocardial SPECT examinations. As myocardial PET is not a service of the statutory health insurance in Germany and is only performed very occasionally, no data were collected on this.

### The key statements from the 2012 to 2021 surveys are as follows:

Since 2012, a long-term positive trend with a continuous growth has been observed. Stress MPS increased by 43%, rest MPS by 41%. A slightly higher growth of 47% was recorded by centres that participated in all surveys. This higher figure is probably a consequence of a selection bias since centres with a cardiac focus are more likely to provide data continuously than others.

In the first COVID-19 pandemic year of 2020, only a slight increase and no decrease in MPS examinations were observed. The NASHIP data from 2021 suggest that the “2020 kink” was compensated in the following year.

Some studies reported a significantly lower number of nuclear medicine procedures and correspondingly of MPS studies in 2020 during the first lock-down restrictions from the COVID-19 pandemic [12–14]. The INCAPS COVID-19 investigators even report an average reduction of 79% in stress SPECT procedures In Europe between March 2019 and April 2020 [15]. According to NASHIP data covering 1 year, the massive lock-down failure was compensated in German in the following months. In a web-based questionnaire with 91 returns, a decrease of “only” 1.4% was found in MPS studies in 2020 compared to 2019 [16]. Our results even document an increase. It is possible that the COVID-19 survey by Freundenberg et al. involved more centres with declining numbers.

The positive trend of the NASHIP data is confirmed by the individual assessment of the participating centres. In the last survey, about 40% recorded an increase, > 70% stable or increasing examination numbers, and only 9% a decrease. The possible cause of the decline cannot be attributed with certainty to a single modality, but lies, as one could expect, in the competitive imaging modalities in general. The COVID-19 pandemic was considered a possible cause of declining investigations in one fifth of the centres (*n* = 4). Since this affected only a few, there was no effect in the total MPS trend.

The most recent data on cardiac MRI and CT examinations in Germany were published for 2019 [17]. In this survey, 69,286 cardiac CT and 64,281 cardiac MRI were registered. A subdivision according to the type of examination was not made. Therefore, the numbers of cardiac CT and MRI examinations for diagnosis of chronic coronary syndrome (CCS) is unknown, but it is likely that they play a minor role. The same rationale applies to the number of stress echocardiographies. All this explains the only weak trend of a shift from MPS to other modalities and confirms that MPS is the leading modality for non-invasive diagnosis of CCS. Accordingly, it becomes apparent that cardiologists are the largest and most constant referral group over the years.

ICA shows a decreasing trend by 12% since 2015. There were in 2021 about 3.2 ICA for every MPS. Even if the other non-invasive imaging modalities are added, the ratio is likely to change only slightly. Thus, a significant proportion of ICA in the context of CCS continues to be performed without prior imaging, contrary to the ESC guideline recommendations [18]. This issue may explain why less than 50% of ICA leads to an interventional therapy. Data from the IQTIG (Institute for Quality Assurance and Transparency in Health Care) indicate a rate of 54.6% in 2015 and of 60.3% in 2019 for non-invasive ischaemia detection during elective coronary angiography [19, 20]. Information on the methods used for ischaemia detection was not given. To summarise the data, there is a great potential for all non-invasive CCS imaging modalities in the future.

The growing number of MPS examinations is accompanied by a continuous centralisation. The average MPS count of centres shows a steady increase during the observation period and is 60% higher than in 2012. At the same time, there is a growing number of centres with a high volume of examinations and a decrease in those with few examinations. Since centres with single-head cameras and those without scoring performed few examinations, the trend towards high-performing centres is encouraging because expertise goes hand in hand with the number of studies and is an indirect indicator of good quality.

Further quality indicators can be derived from the procedural and technical data. In terms of camera equipment, a shift towards more SPECT-CT systems and a slight increase in CZT and other dedicated cardiac cameras is found. As a result of more SPECT-CT cameras, more MPS with attenuation correction are performed. Together with the high number of gated SPECT acquisitions, a prerequisite for high quality results is given [21]. In terms of reporting, there is still a weakness and obvious need for training in the use of perfusion scores, even though there has been a positive development over the observation period. A utilisation rate of 100% remains desirable.

Stress testing as one essential element of every MPS examination changed to pharmacological stress tests. In 2021, exercise stress was no longer the most common stress test and had been replaced by regadenoson and adenosine, with regadenoson as the most common pharmacological stress agent. The decline in ergometry was greatest from 2018 to 2021 and is related to a shift to more pharmacological stress due to the COVID-19 pandemic, but also reflects increasing amounts of patients with limiting comorbidities.

In terms of protocols, thallium-201 has been abandoned due to its high radiation dose and guideline recommendations [22, 23]. With regard to radiation dose, the use of 2-day protocols and stress-only protocols is welcome. Especially in hospitals, 1-day protocols are used due to the high time pressure in order to reduce long hospital stays.

## Conclusion

The 2012 to 2021 MPS surveys reveal a continuously growing number of examinations with only a mild effect of the COVID-19 pandemic and a centralisation of MPS imaging with increasing numbers per centre. In Germany, MPS is the leading non-invasive imaging modality in CCS. Performance and technical data reveal a high-grade adherence of routine MPS practice to current guidelines. Potential in training remains in the field of scoring and relates mainly to centres with low MPS numbers. Relating MPS numbers and the available numbers of the other imaging modalities to ICA, a large potential of non-invasive diagnostics in CCS remains in the future.

## Data Availability

The data on which this article is based are not publicly available in order to protect the privacy of the departments submitting their data. This was explicitly promised to all participants. Data can be passed on anonymously by the corresponding author on reasonable request.
